# No Weight Catch-Up Growth of SGA Infants Is Associated with Impaired Insulin Sensitivity during the Early Postnatal Period

**DOI:** 10.1155/2010/704642

**Published:** 2010-12-08

**Authors:** Tong-yan Han, Xin-li Wang, Yun-pu Cui, Hong-mao Ye, Xiao-mei Tong, Mei-hua Piao

**Affiliations:** Department of Pediatrics, Peking University Third Hospital, Beijing 100083, China

## Abstract

*Objective*. To investigate the relationship between weight catch-up growth and insulin sensitivity in small for gestational age (SGA) infants. *Methods*. Forty-four singleton SGA subjects met the inclusion criteria and finished-3-month followup. Body weight, length, fasting glucose, and fasting insulin (FI) levels were measured at 3 days and 3 months. Insulin sensitivity was evaluated by FI and homeostasis model assessment (HOMA). *Results*. According to the change of weight Z-score, forty-four subjects were divided into two groups: noncatch-up growth (NCUG) and catch-up growth (CUG). By 3 months of age, the body weight, body length and BMI of NCUG group were significantly lower than those of CUG group. The FI and HOMA were significantly higher in NCUG group. The change of weight Z-score during 3 months was inversely related to the HOMA at 3 months. *Conclusion*. Our data exemplified that no weight catch-up growth during the first 3 months was associated with impaired insulin sensitivity in SGA infants.

## 1. Introduction

The term “small for gestational age” (SGA) is sometimes used synonymously with low birth weight, but actually it refers to a low birth weight with respect to gestational age read on references curves and not a low birth weight per se. During the past decades, dozens of epidemiological studies associated with low birth weight infants confirmed programming hypotheses first proposed by Barker et al. in 1989 [1]. During critical windows of intrauterine development, some deleterious stimuli, such as undernutrition or alterations in placental function, may induce permanent changes in cell/tissue structure and/or function. It indicates that factors distinct from gene inheritance can be modulated by unique events during the life of an individual and lead to permanent changes. Subsequently, it has been suggested that such “programming” events are not restricted to fetal life but may occur during several critical windows during development [2], such as the early postnatal period.

Catch-up growth is the acceleration in growth of 85%–90% SGA infants soon after birth [3]. Maximum catch-up growth usually occurs in the first 6 months of life but may continue up to 2 years [4]. In more than 80% of infants born SGA, catch-up growth occurs during the first 6 months of life. For this reason, growth monitoring during the early postnatal period provides useful information, and different growth patterns may be identified in infants as young as 3 months of age. Rapid weight gain, or “catch-up”, was associated with a lower risk for hospital admission and lower mortality for SGA. There is a concern, however, that although accelerated postnatal weight gain may benefit the child, by improving his or her nutritional status, resistance to infection, and survival, there could be a cost to be paid later, in the form of obesity [5], impaired glucose tolerance in young adults [6], and increased mortality from coronary heart disease [7]. 

The aim of this study was to examine the relationship between the weight catch-up growth and insulin sensitivity in SGA infants during the first 3 months of life.

## 2. Subjects and Methods

### 2.1. Subjects

The subjects in our study were recruited from singleton newborns that were delivered from June through December 2009 in the department of obstetrics and subsequently followed in the department of pediatrics of the Third Hospital, Peking University. 

 Neonates were included in this study if they met the following inclusion criteria: (1) they had experienced a normal pregnancy with gestational age of >33 weeks; (2) their birth weights were below the 10th percentile of the sex-specific distribution for gestational age using birth weight standards of Chinese; (3) they had a 1-minute Apgar score of >7 and a 5-minute Apgar score of 10. 

 We excluded infants born to women with diabetes, gestational diabetes, or chronic hypertension, and infants who had intrauterine infections, congenital malformations, and major neonatal problems. We also excluded the infants who were not breastfed from birth to 3 months. 

A complete record with parental, pregnancy, and perinatal information was filled at entry. Clinical data were collected prospectively. These included gestation, mode of delivery, birth weight, and length and Apgar scores of 1 minute and 5 minutes.

The study protocol was approved by the Ethics Committee of Third Hospital, Peking University. Subjects' parents gave informed written consent before enrollment. The investigation conformed to the principles outlined in the Declaration of Helsinki as revised in 2000.

### 2.2. Methods

#### 2.2.1. Anthropometric Measurements

Midwives measured the birth weights and crown-heel lengths within 2 hours of delivery. Birth weights were recorded to the nearest gram using a balance scale. The crown-heel lengths were measured with a length board by the standard anthropometric technique. A single neonatal pediatrician performed gestational age assessment on each study participant according to the Dubowitz Scoring System within 48 hours of birth. 

Infancy weight and length were measured by standard clinical procedures during routine visits at Pediatric Department at age of 3 months (with corrected age for preterm infants).

#### 2.2.2. Feeding Method

Subjects were breastfed from birth until 3 months.

#### 2.2.3. Fasting Glucose and Insulin Concentrations

Blood was obtained by heel prick before feeding between 7:00 and 9:30 AM (≥2 hours' fast) on 3 days post of delivery and 3 months of age (with corrected age for preterm infants) and analyzed for fasting glucose (FG) and fasting insulin (FI) concentration. 

Glucose concentrations were measured by using the SureStep Plus System from LifeScan (Milpitas, CA). Interassay and intra-assay coefficients of variation for glucose were 0.9% and 1.8%, respectively. Insulin was measured by enzyme-amplified immunoassay using active insulin ELISA Kit (DSL-10-1600; Diagnostic Systems Laboratories, Webster, TX). The detection limit of this assay was 0.26 *μ*IU/mL (1.81 pmol/L) in our laboratory, and the intra- and interassay coefficients of variation were 2.6% and 5.2%, respectively.

#### 2.2.4. Calculation

Catch-up growth outcomes in 3 months were described using the standard deviation (SD) scores, or Z-scores, calculated from the normal population of the same chronological age and gender of Chinese. Weight Z-score were calculated according to the following equation: weight Z-score = (observed value−mean value)/SD. The Z-scores of weight was calculated at birth and 3 months of age (with corrected age for preterm infants). 

The previously validated homeostasis model assessment (HOMA) was used to estimate insulin sensitivity [8], with higher HOMA that indicates more impaired insulin sensitivity. HOMA was calculated from the fasting glucose and insulin concentrations according to the equation: HOMA = [insulin (*μ*U/mL) × glucose (mmol/L)]/22.5]. Birth size and shape measures were birth weight, birth length, and ponderal index (PI = [birth weight (g)/birth length (cm)^3^] × 100) [9], studied as continuous variables. Body mass index (BMI) was used as a measure of relative adiposity and was calculated according to the following formula: BMI = weight (kg)/length (m)^2^ [10].

#### 2.2.5. Evaluation of Weight Catch-Up Growth

The weight catch-up growth was evaluated by the change between the weight Z-scores at birth and at 3 month. The change of weight Z-score during the 3 months less than (or equal to) 0 Z-score was defined as noncatch-up growth (NCUG) and that greater than 0 Z-score was defined as catch-up growth (CUG).

#### 2.2.6. Statistical Analysis

The data were expressed as mean ± SD. Differences between the 2 groups were compared by unpaired Student's *t-*test and Pearson *χ* ^2^ test. FI and HOMA were logarithmically transformed (log _10_ ) before the analysis to approach normal distribution. The relationship between the change of weight Z-score and logarithmically transformed HOMA was evaluated by Pearson correlation. *P* < .05 was considered statistically significant. All statistical analyses were performed using the Statistical Package for Social Science program (SPSS for Windows, version10.0; SPSS, Chicago).

## 3. Results

Parents of sixty-two SGA infants gave initial consent, and blood samples were obtained in 58 at birth. Fifty-four collections met the above inclusion criteria, and forty-four subjects finished the 3-month followup, thus were taken into the scope of the study. They had no major neonatal problems and had normal acid-base status at birth. There was no history of maternal hypertension, diabetes, or infections. Mean gestational age and birth weight of the study population were 36.46 ± 2.38 weeks and 1996.59 ± 353.15 g, respectively. The male/female ratio was 22 : 22. Because there were no significant differences between the two genders, we combined the data into one group. 

All 44 SGA infants were divided into two groups according to our criteria of weight CUG: noncatch-up growth (NCUG) group (*n* = 12) and catch-up growth (CUG) group (*n* = 32). Demographic characteristics of gender, maternal age, gestational age, delivery method, and 1-minute Apgar score were not different between two groups (Table 1). There were no statistically significant differences in birth size and shape between NCUG and CUG groups. Furthermore, at 3 days of life, the FG, FI, and HOMA (logarithmically transformed) were not significantly different (Table 2).

As shown in Table 3, by 3 months of age, the body weight, body length, and BMI of NCUG group were significantly lower than those of CUG group. The FI and HOMA (logarithmically transformed) were significantly higher in NCUG group than CUG group, which suggested impaired insulin sensitivity. 

**Figure 1:**
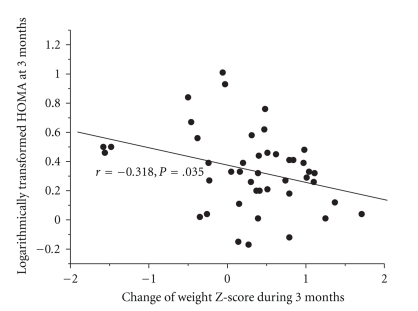
Correlation between change of weight Z-score during 3 months and logarithmically transformed HOMA at 3 months of age. The change of weight Z-score during 3 months was inversely related to the HOMA (logarithmically transformed) at 3 months (*r* = −0.318; *P* = .035).

In SGA infants, the change of weight Z-score during 3 months was inversely related to the HOMA (logarithmically transformed) at 3 months (*r* = −0.318; *P* = .035) (Figure 1).

## 4. Discussion

This study presents data on the development of insulin sensitivity in term and near term SGA infants from birth to 3 months. There were significant differences in weight, length, BMI, fasting insulin, and HOMA between the SGA infants with and without weight catch-up growth, and no weight gain was inversely related to HOMA by 3 months. We found that if there was no gain in weight SD score during the first 3 months of SGA infants, their insulin sensitivity, which was assessed using fasting insulin, fasting glucose, and HOMA, was relatively impaired during this period. 

We chose to study SGA infants because this is one human population subject to early undernutrition and consequently marked variation in early postnatal growth. SGA infants recruited to this study were born at term or near term, had normal acid-base status at birth, no history of maternal hypertension, and were breastfed during the study. The data were, therefore, not confounded by factors that were reported previously to be associated with serum insulin. 

The present study has certain limitations. Our relatively small sample size and short duration of followup reduced the power to robustly predict impaired insulin sensitivity related to weight catch-up growth. Furthermore, our study was restricted to SGA infants who were born in our hospital. Selection bias may have occurred to the extent that only babies born in the hospital were studied. 

The relationship between insulin sensitivity and catch-up growth of weight remains controversial. Singhal et al. found that later insulin resistance was greatest in infants born preterm with accelerated growth in the first 2 weeks [11], the period of fastest postnatal growth, and least in those who grew poorly. Ekelund et al. reported that rapid weight gain from birth to six months was associated with metabolic risk factors at 17 years of age [12, 13]. Moreover, Hales et al. suggested that rapid catch-up growth during the first year postnatal specifically impairs insulin secretion and action [14]. Soto et al. studied 85 SGA infants and 23 AGA infants at 1 year of age and found that catch-up growth in weight until the age of 1 year was associated with higher fasting insulin levels [15].

On the contrary, Barker et al. found that raised fasting plasma insulin and proinsulin concentrations, two measures of insulin resistance [16], were associated with low birth weight and low BMI at 2 years of age. Furthermore, in a detailed retrospective longitudinal study of 8760 children who were born in Helsinki during 1934–44 with records of their monthly changes in body size from birth to 2 years of age [17], the boys who later developed coronary heart disease tended to be thin at birth and to have low weight gain in infancy. These observations are consistent with findings among men in Hertfordshire, where low weight at-age-1 year similarly added to the increased risk of coronary heart disease associated with low birth weight [1]. Although these studies include infants across the normal range of birth weights rather than focusing on SGA infants, they nevertheless are consistent with the observations in the current study. 

Our finding suggests that noncatch-up growth during the first 3 months may have adverse long-term implications for insulin resistance. One possible mechanism exists by which reduced fetal and infant growth and accelerated weight gain after 1 or 2 years may lead to insulin resistance. SGA infants who are thin at birth lack muscle, a deficiency which will persist, as there is little cell replication after birth [17]. They complete their catch-up growth and weight gains between birth and age of 2 years but remain thin at 2 years of age [6], showing greater accumulation of total body and abdominal fat afterwards [18]. If they develop a high BMI in childhood, they may have a disproportionately high fat mass. This may be associated with the development of insulin resistance, as children and adults who had low birth weight but are currently heavy are insulin resistant.

In conclusion, the present study demonstrates that SGA infants without weight catch-up growth during the first 3 months of life present relatively impaired insulin sensitivity. Although the role of early postnatal growth from birth to two years of age remains unresolved, our current study provides much more precise information on the timing of the weight gain that appears to be related to later health. Because of the limitation of the study, we believe that a larger sample size with more AGA infants as a control group addressing the impact of catch-up growth on insulin sensitivity could provide a more robust result.

## Figures and Tables

**Table 1:** Comparisons of demographic characteristics between NCUG and CUG groups.

	NCUG	CUG	*t*/*χ* ^2^	*P*
	*n* = 12	*n* = 32		
Maternal age, y^a^	29.30 ± 6.65	29.32 ± 2.76	0.014	.989
Maternal height, m	1.62 ± 0.71	1.63 ± 0.75	0.254	.801
Maternal weight, kg	58.67 ± 4.98	60.13 ± 6.07	0.742	.462
Maternal BMI	22.28 ± 1.58	22.62 ± 1.63	0.623	.537
Male, n (%)^b^	7 (58.3%)	15 (46.9%)	0.458	.498
Vaginal delivery (*n*, %)^b^	7 (58.3%)	18 (56.3%)	0.015	.901
Gestational age, (wk)^a^	35.58 ± 2.15	36.78 ± 2.41	1.511	.138
Apgar score at 1 min	9.17 ± 0.94	9.25 ± 1.05	0.241	.810

Data are expressed as the mean ± SD or number (%).

Comparisons were performed with ^a^unpaired Student's *t *test or ^b^Pearson's *χ* ^2^.

**Table 2:** Comparisons of birth size and shape and metabolic characteristics at 3 days of life between NCUG and CUG groups.

	NCUG	CUG	*t*/*χ* ^2^	*P*
	*n* = 12	*n* = 32		
Weight (kg)	1855.83 ± 338.91	2049.38 ± 348.84	1.651	.106
Weight (SDS)	−1.99 ± 0.53	−2.15 ± 0.54	0.890	.378
Length (cm)	42.92 ± 2.57	44.13 ± 2.79	1.305	.199
Length (SDS)	−1.81 ± 0.95	−2.00 ± 0.96	0.606	.548
PI (g/cm^3^)	2.28 ± 0.34	2.39 ± 0.27	0.992	.328
FG (mmol/L)	3.68 ± 0.91	4.12 ± 0.93	1.413	.165
FI (mIU/L)	11.08 ± 9.60	12.68 ± 9.79	—	—
log _10_ FI	0.91 ± 0.38	0.98 ± 0.34	0.619	.539
HOMA	1.86 ± 1.71	2.39 ± 2.19	—	—
log _10_ HOMA	0.11 ± 0.42	0.23 ± 0.37	0.933	.356

Data are expressed as mean ± SD. FI and HOMA were logarithmically transformed (log _10_ ) before the analysis to approach normal distribution. All statistical comparisons were performed with unpaired Student's *t* test—indicates not applicable.

**Table 3:** Comparisons of body size and shape and metabolic characteristics at 3 months of age between NCUG and CUG groups.

	NCUG	CUG	*t*	*P*
	*n* = 12	*n* = 32		
Weight (kg)	4753.33 ± 137.33	5403.44 ± 385.78	8.241	.000
Weight (SDS)	−2.42 ± 0.55	−1.51 ± 0.49	5.333	.000
Length (cm)	56.46 ± 1.31	58.14 ± 1.11	4.272	.000
Length (SDS)	−3.00 ± 0.58	−1.83 ± 0.69	5.247	.000
BMI (kg/cm^2^)	14.92 ± 0.55	15.98 ± 1.01	4.412	.000
FG (mmol/L)	4.18 ± 0.58	4.32 ± 0.64	0.636	.528
FI (mIU/L)	21.92 ± 14.26	10.76 ± 4.69	—	—
log _10_ FI	1.25 ± 0.30	0.99 ± 0.19	3.422	.001
HOMA	4.15 ± 2.96	2.11 ± 1.06	—	—
log _10_ HOMA	0.52 ± 0.32	0.27 ± 0.22	2.913	.006

Data are expressed as mean ± SD. FI and HOMA were logarithmically transformed (log _10_ ) before the analysis to approach normal distribution. All statistical comparisons were performed with unpaired Student's *t *test—indicates not applicable.

## Abbreviations

SGA: Small for gestational age
HOMA: Homeostasis model assessment
NCUG: Noncatch-up growth
CUG: Catch-up growth
AGA: Appropriate for gestational age
PI: Ponderal index
BMI: Body mass index.
